# Players and processes behind the national health insurance scheme: a case study of Uganda

**DOI:** 10.1186/1472-6963-13-357

**Published:** 2013-09-22

**Authors:** Robert K Basaza, Thomas S O’Connell, Ivana Chapčáková

**Affiliations:** 1 Planning Department, Ministry of Health Uganda, P.O. Box 27450, Kampala, Uganda; 2 Institute of Health, Policy and Management, International Health Sciences University, P.O. Box 7787, Kampala, Uganda; 3 Health Section, Programme Division, United Nations Children’s Fund, 3 UN Plaza, New York, NY 10017, USA

**Keywords:** Health insurance, Stakeholder analysis, Context analysis, Policy reform, Health financing, Case study, Uganda

## Abstract

**Background:**

Uganda is the last East African country to adopt a National Health Insurance Scheme (NHIS). To lessen the inequitable burden of healthcare spending, health financing reform has focused on the establishment of national health insurance. The objective of this research is to depict how stakeholders and their power and interests have shaped the process of agenda setting and policy formulation for Uganda’s proposed NHIS. The study provides a contextual analysis of the development of NHIS policy within the context of national policies and processes.

**Methods:**

The methodology is a single case study of agenda setting and policy formulation related to the proposed NHIS in Uganda. It involves an analysis of the real-life context, the content of proposals, the process, and a retrospective stakeholder analysis in terms of policy development. Data collection comprised a literature review of published documents, technical reports, policy briefs, and memos obtained from Uganda’s Ministry of Health and other unpublished sources. Formal discussions were held with ministry staff involved in the design of the scheme and some members of the task force to obtain clarification, verify events, and gain additional information.

**Results:**

The process of developing the NHIS has been an incremental one, characterised by small-scale, gradual changes and repeated adjustments through various stakeholder engagements during the three phases of development: from 1995 to 1999; 2000 to 2005; and 2006 to 2011. Despite political will in the government, progress with the NHIS has been slow, and it has yet to be implemented. Stakeholders, notably the private sector, played an important role in influencing the pace of the development process and the currently proposed design of the scheme.

**Conclusions:**

This study underscores the importance of stakeholder analysis in major health reforms. Early use of stakeholder analysis combined with an ongoing review and revision of NHIS policy proposals during stakeholder discussions would be an effective strategy for avoiding potential pitfalls and obstacles in policy implementation. Given the private sector’s influence on negotiations over health insurance design in Uganda, this paper also reviews the experience of two countries with similar stakeholder dynamics.

## Background

This paper is a contextual and stakeholder analysis of formulating and setting policies for a proposed National Health Insurance Scheme (NHIS) in Uganda. It underscores the lessons learnt over the last decade with regard to the upcoming Ugandan scheme and those of other low- and middle-income countries considering NHIS. Of the five East African countries, only Uganda is without National Health Insurance (NHI) [1]. The establishment of NHI policies is frequently linked to a change in a country’s political climate, as in the case of Ghana [2,3] and Tanzania [4]. Insurance can also overcome regressive aspects related to user fee policies [2,5]. On both counts, Uganda is an anomaly. First, the drive for a health insurance policy has been sustained by the ruling National Resistance Movement (NRM), in power for over 25 years, and this has contributed to a strong sense of government ownership and deep institutional knowledge. Second, Uganda abolished formal user fees in 2001 in all public health units (with the exception of private hospital wings) to eliminate financial access barriers [6]. Nonetheless, Ugandans have continued to experience high levels of Out-Of-Pocket (OOP) expenditure (50%) [7] owing to indirect fees (such as transportation costs), supplementary fees to pay for medicines and supplies from private vendors, and illegal fees demanded by medical staff for purportedly free services [8]. The proposed Uganda NHIS is to be a contributory health financing mechanism, in which members pay a premium in exchange for a defined package of services, containing elements from both formal and informal employment sectors. The scheme shall be established by an act of parliament as part of proposed reforms to achieve universal health coverage. The NHIS has been promoted to mitigate regressive OOP spending and offer a more effective policy path for this country of 34 million people to have equitable access to universal healthcare [9].

The first part of the paper provides the following: (1) a brief recapitulation of the socio-economic and political context underlying the development of the NHIS; and (2) a process analysis of the three phases of health insurance development and contextualised stakeholder management. The second part is a stakeholder analysis that covers the following: (i) a description of the methods used for the stakeholder analysis; (ii) a categorisation of the key policy players; (iii) a discussion of the process of stakeholder engagement and management strategies employed by the government; and (iv) a conclusion with lessons relevant to policy makers on how a stakeholder-driven and broadly inclusive policy development process can increase the likelihood of policy implementation.

### Socio-economic and political context underlying NHIS development

Uganda is a low-income country with a Gross Domestic Product (GDP) of US$550 per capita in 2012. The proportion of the population employed in the informal sector was 54.5% and 14.7% of the population was urbanised in 2012 [10]. Over the 2000–10 period, Uganda experienced an annual average economic growth of 7% [11]. Nonetheless, an estimated 24.5% of the population was living below the $1-per-day poverty line, and more households became impoverished as a result of health expenditure on a daily basis [11]. Despite significant progress towards the Millennium Development Goals (MDGs), it is unlikely that MDG 4 (child survival) and MDG 5 (maternal health) will be met [12]. A lack of financial access to health services, the resultant poor health, and the high disease burden of the poor have been identified as drivers of poverty in Uganda [13], which demonstrates the importance of minimising the financial barriers to health services.

Of the population, 72% resides within a 5-km radius of a health facility, and universal primary and secondary education has boosted literacy rates. The average national literacy rate in 2010 was 76%. Progress in overall human development indicators in Uganda has been consistent but relatively low. The life expectancy improved from 45 years in 2003 to 52 years in 2008. Key health impact indicators, particularly infant and under-5 mortality, are improving [14]. However, maternal and child death remain high, accounting for 20.4% of the disease burden in the country. In 2010, the maternal mortality ratio was estimated at 435 deaths per 100,000 live births and the infant mortality rate was estimated at 54 deaths per 1,000 live births. Uganda faces a double epidemic of communicable and non-communicable diseases. Communicable diseases account for 54% of the total burden of disease in the country, with HIV/AIDS, tuberculosis, and malaria being the leading causes of ill health. Non-communicable diseases are an emerging problem with costly treatment implications. Coupled with this is the high fertility rate, which at 6.2 per woman is the second-highest rate in the world. In addition, significant disparities exist in health status among regions and socio-economic strata, with rural areas having the highest burden of ill health and death [10,15]. On the political front, Uganda has progressed towards a multi-party democracy. The stability of the country’s political system can be seen as offering a strong advantage for establishing the NHIS. Having held office for 26 years, President Museveni of the NRM is one of the longest-serving leaders in sub-Saharan Africa. The impetus for the NHIS is strongly associated with democratic reforms in Uganda, where the NRM won the first multi-party elections in 2006 and again in 2011.

### Financing health services in Uganda

In 2001, Uganda pledged to increase national spending on health to at least 15% of the national budget—in alignment with the Abuja Declaration at the summit of African Heads of State [16,17]. Since then, government health expenditure as a percentage of total government expenditure has remained under 10%: it was 7% in 2010. The Total Health Expenditure (THE) per capita in 2010 was $52, which was 9% of nominal GDP. The government health expenditure was 22% of the THE, which was US$11.2 per capita per annum. Household expenditure on health was 42% of the THE, and the balance of 36% came from donors and NGOs. Also in 2010, household OOP spending on health per capita was US$22. In terms of financing sources as a percentage of THE, private spending provides 49%, donors and non-governmental organisations (NGOs) 36%, and public (government) 15% [18]. The proportion of households that incurred catastrophic health expenditure was 28%, concentrated mostly in poor households [19]; 2.3% of all households were newly impoverished as a result of OOP spending in 2006 [20]. Overall, health-sector financing is project and vertically oriented, with low investments in overall system improvement [21]. The incidence of catastrophic health expenditure among the poor steadily increased from 1996 to 2006 despite the abolition of user fees, which was partly due to the greater use of private providers by the poor [8,22]. Another factor was medicines frequently being unavailable in public facilities, obliging patients to pay higher prices to acquire medicines at private pharmacies [23]. There is also a lack of a comprehensive social protection strategy in Uganda [20]. Current protection is minimal, with Community Health Insurance (CHI) schemes being accessible to just 5–10% of the population in the few areas where such schemes operate [6]. Private commercial health insurance schemes cover only an additional 1% of the population and are mainly limited to the in-house private health maintenance plans of a few large firms plus some third-party insurance companies [24]. With no social health-protection mechanism currently in place and soaring OOP health costs, the development of an equitable, sustainable health-financing mechanism for the NHIS is seen as a key strategy to achieve the MDGs [25]. Table 1 summarises the key government policies to reduce poverty and improve health and their relevance to the evolution of the current version of the proposed NHIS.

**Table 1:** Key policies relevant to the proposed Uganda NHIS

**Policy**	**Provisions relevant to NHIS**
Constitution of Uganda 1995 [26]	• Objective XIV (b) is to ensure that Ugandans have access to health services
	• Objective XX is a commitment to take all measures to provide basic medical services to the population
Health Sector Strategic Plan (HSSP) 2000/01–2004/05 [27]	• SHI as one of the key objectives of its healthcare financing component
	• Government would continue to develop and support alternative healthcare financing schemes
Poverty Eradication Action Plan (PEAP) 2004/2005–2007/2008 [28]	• Objectives are pro-poor focused and are consistent with the MDGs
	• Human development and improving health outcomes among the key priorities
Cabinet Minute No. 63 (CT 2006)	• Directed the Minister of Health to issue drafting instructions for the bill establishing the National Social Health Insurance Scheme to the First Parliamentary Council; Directorate in the Ministry for Justice responsible for legislative drafting
National Development Plan (NDP) 2010/11 – 2014/15 [29]	• Provides for the establishment of health-financing mechanisms (NHI and other community health-financing mechanisms) based on prepayment and financial risk pooling with the goal of achieving universal coverage and social health protection
NRM Presidential Manifesto 2011–2016	• Provides for establishing a national health insurance scheme as one of the work programs in the health sector in 2011–16
Health Sector Strategic and Investment Plan (HSSIP) 2010/11–14/15 [30]	• Calls for health insurance, with the goal to increase financial access to healthcare and reduce the catastrophic expenses that impoverish households, to be introduced gradually, leading eventually to universal health coverage
	• Commitment to fully harmonise health policies, standards, and guidelines for the East African Community by 2014–15
	• Guided by the principles of: (i) access for all to a minimum package of services; (ii) equitable distribution of services; and (iii) effective and efficient use of health resources
National Health Policy [14]	• Upholds the obligation of the government regarding citizens’ access to healthcare
	• Guided by the same principles as HSSIP 2010/11–14/15
Cabinet Minute No. 84 (CT 2011)	• Directed the Minister of Health to issue additional principles of the bill to the First Parliamentary Council taking into consideration stakeholder concerns

### Process of NHIS development

The development of the NHIS took place in three phases: phase I, from 1995 to 1999; phase II, from 2000 to 2005, and phase III, from 2006 to 2011. These phases are based upon key events linked to the health sector and the national 5-year planning frameworks.

### Phase I, 1995–99: ‘people’s power’

This period was characterised by decentralisation, the introduction of user fees, and CHI schemes, and we label this as ‘people’s power’. During this period, people made an effort to engage in decisions that governed their lives; the period saw the introduction of health-financing reform and broad decentralisation. The passage of the 1997 Local Government Act decentralised governance and delivery of health services. In 1996, Uganda’s Ministry of Health (MOH) commissioned the first feasibility study on health insurance, and some limited CHI pilot schemes were attempted [31]. The feasibility study was exploratory and looked at the potential for establishing social health insurance in Uganda. The study pointed out the limited knowledge that existed in Uganda with regard to social health insurance policies and administration. It recommended a further detailed examination of implementation arrangements [32]. The first Poverty Eradication Action Plan, which was finalised in 1997, established a participatory national development strategy based upon pro-poor and poverty-reduction policies, including pro-poor budget allocation [33]. These policies led to the National Health Policy (1999–2009); its objectives included universal coverage to achieve improved health outcomes and equity, and it was financed through a countrywide Social Health Insurance (SHI) model [34]. Subsequently, the government launched the Health Sector Strategic Plan 2000–05, which increased spending on health coupled with higher user fees and was introduced in 1993 as a precondition for a World Bank loan [35]. Shortly after, a presidential order in March 2001 abolished user fees, having been driven by both a 1999 World Bank report on the regressive impact of user fees on healthcare access and the desire to bolster re-election chances in the upcoming 2001 presidential elections [35,36]. The years 1997 to 2001 represent a period during which differences of opinion among the key internal stakeholders within the MOH on the proposed SHI model intensified [31]. Moat and Abelson pointed out that the ‘big man’, the president, abolished user fees without due consultation among key stakeholders and clients, including civil society, the managers of public health facilities, and private providers [37].

### Phase II, 2001–05: ‘hopeful start’

This was a phase of SHI design that we describe as a ‘hopeful start’. Continued poor financing of health-system access led the government to commission a second feasibility study on health insurance. This aimed to align the design of health insurance with the first health policy, the Health Sector Strategic Plan of 2000–05, and to provide insight into implementation plans [32]. Specific recommendations included phased SHI implementation starting with the formal sector; with time, this would expand to informal-sector protection [38]. The result was the establishment in 2005 of the SHI Secretariat by the MOH. A cabinet paper produced by this secretariat in 2006, ‘Principles of SHI’, was circulated to stakeholders, including other government ministries, professional medical associations, trade unions, and local government associations for sensitisation and consultation [39]. The MOH also undertook visits to Thailand, India, and Tanzania to study models of national insurance, and conducted additional studies with Providing for Health (P4H), an alliance of four European governments (Germany, Switzerland, Spain, and France); two multilateral organisations (the World Bank and African Development Bank); and two United Nations agencies (International Labour Organization and the World Health Organization [WHO]), and others. Following a 2-year consultative process, the updated principles of SHI were presented to the cabinet and adopted in 2006 [20]. The Minister of Health was directed to prepare a bill on establishing SHI and initiate the legislative process for introducing SHI.

### Phase III, 2006–11: ‘stakeholder concerns’

We categorise this period as ’stakeholder concerns’. Following resistance to the bill to establish SHI, the MOH set up a multi-sectoral working group, the National Task Force (NTF), to spearhead a participatory design process for drafting a revised insurance bill. An attempted stakeholder analysis was undertaken to help identify issues relevant to the design of the health insurance scheme; however, the analysis had minimal utility since there was an apparent lack of experience on the part of the working group in carrying out and using stakeholder analysis for policy development. Furthermore, this attempt at stakeholder participation was not genuinely broad based. The National Social Security Fund (NSSF) did not join the health insurance scheme despite an appeal by the NTF for it to do so [20]. One factor was that such stakeholders as the NSSF perceived health insurance to be a project of the MOH rather than a broad-based government initiative [40]. The NTF produced a revised SHI bill in 2008 as well as a framework of its organisational aspects and the accreditation of providers.

Though the NTF succeeded in bringing together public and private sectors in a common forum, it did not effectively create ownership among the private-sector players or engage in a redesign of the proposed scheme. Instead, the NTF sought to prioritise the sensitisation and buy-in of stakeholders by advocating the existing SHI framework [20]. As a result, the proposed SHI plan still lacked the backing of some major stakeholders—notably private-sector employers and employees. In early 2009, the NTF held workshops for the cabinet and parliament, which prompted the government to request technical support from P4H. The P4H assessment identified low levels of stakeholder involvement as an impediment to implementing SHI, and it recommended that their engagement be significantly reinforced [20]. The assessment also recommended greater inter-ministerial coordination as well as openness, clarity, and public engagement with respect to the SHI and—importantly—to its alternatives. Interviews with stakeholders from the NTF revealed that the majority of the task force members supported the general idea of SHI, but they felt that the plan lacked clarity with regard to efficiency, effectiveness, and supervision. NTF stakeholders also questioned the equity of a phased SHI implementation according to employment group [19,20].

The recommendations of P4H and other development partners led in October 2009 to the prime minister creating a cabinet sub-committee composed of four state ministers: health; finance, planning, and economic development; public service; and labour. The drafting committee was expanded to include public- and private-sector experts, such as the Insurance Regulatory Authority of Uganda, private insurers, and representatives of CHI schemes, so as to broaden the ownership and buy-in.

In 2010, a review by the MOH and WHO explored feasible health-financing options for health insurance. By mid-2011, the cabinet sub-committee redrafted the SHI principles. It started by successfully negotiating a name change from an SHI plan, which implied that it alone could potentially cover all citizens, to an NHIS, which signalled the use of multiple insurance component plans to cover various segments of the population. This was strongly endorsed by private insurance providers, which were concerned about market displacement because it preserved their existing market of private-sector firms [41]. The sub-committee also secured a consensus on establishing a solidarity fund with tariffs and rates for healthcare, greater market liberalisation for the provision of health insurance, and adding a CHI component to cover individuals working in the informal sector. The private sector was reassured that its commercial health insurance schemes and third-party health administrators would not face competition from the NHIS because the SHI component was limited to targeting public-sector workers and the CHI pertained to the informal sector. Though direct presidential endorsement was not provided, the cabinet approved these revised principles in September 2011, with the expectation that the NHIS scheme would be introduced in 2013 [42].

### Financing the proposed NHIS

NHI can improve overall health financing if it ‘raises adequate funds for health, in ways that ensure people can use needed services, and are protected from financial catastrophe or impoverishment associated with having to pay for them’ [43]. Diverse sources of healthcare funding are often cited as useful for achieving a stable health financing system [44], and a mixed financing approach is common in Africa [45,46]. It was proposed that the planned NHIS be financed by 4% of payroll deductions from employees with an additional 4% from employers [47]. The plan calls for a solidarity fund, into which annual contributions from the government, donors, and the formal sector will pay a portion of their premium to cover indigent (including unemployed) persons—currently estimated at 25% of the population [33]. Formal-sector workers and up to four of their dependents—accounting for approximately 10% of the country’s population—will be eligible to join the Social Health Insurance Scheme and Private Commercial Health Insurance Schemes at the inception of the NHIS. The informal-sector workers and their dependents will join the Community Health Insurance Scheme, and in this way the NHIS will progressively cover the entire population.

The above percentage contributions were calculated in a feasibility study by Harvard University and Makerere University Schools of Public Health in 2001 [38] and in 2008 by the WHO/MOH using SimIns (health insurance software). The NHIS will initially generate 153.183 billion shillings in 2013, gradually increasing to 675.552 billion shillings by 2018 (2012 exchange rate: 2,500 shillings per 1 US$). The scheme will initially enrol public formal-sector employees and their dependants and generate more money as enrolment rises among both the informal and formal private sectors workers and their dependants. Virtually all in-patient and out-patient care provided within Uganda will be covered by NHIS. Further details of the benefit package are provided for in a schedule in the draft NHIS bill [47]. The mechanisms for coping with the envisioned increase in patient load in accredited health units and expansion of services in the underserved areas once the scheme begins have yet to be worked out. Table 2 summarises the current components of the proposed NHIS.

**Table 2:** Relationship of NHIS*, Community Health Insurance Scheme, and Private Commercial Health Insurance Scheme

	**NHIS**	CHIS*	PCHIS*
**Common arrangements**			
Role of proposed Board of Directors of NHIS	For oversight of health insurance schemes and in-house operations of public sector workers and their dependants scheme, the social health insurance scheme	Represented on the Board. The NHIS Board will provide regulations	Represented on the Board. The NHIS Board will provide regulations
Regulation by Insurance Regulatory Authority of Uganda.	Yes	Yes	Yes
Participation in solidarity funds	Provides funds to CHI for indigents	Membership for indigents shall be paid by the NHIS	Contributes part of the premium to the solidarity fund for paying premiums to indigents.
**Specific issues**			
Ownership	Government	Private	Private
Current coverage	-	5–10% of the population where the schemes exist	1% of the national population
Target membership	Public formal-sector workers and their dependents	Informal-sector workers and their dependents.	Employees and dependents from the private formal sector
Proposed/current source of funding	Mandatory payroll deductions and contributions from both employees and the government	Contributions from personal earning for those above the poverty line.	Mandatory payroll deductions and contributions from employees and private-sector employers
		Payment from the solidarity fund for those below the poverty line	
Benefit package	Stipulated in the bill	Negotiated with the private healthcare providers	Negotiated with employers, trade unions, and individual members and insurance companies.

*NHIS: National Health Insurance Scheme;

*CHIS*: Community Health Insurance Scheme;

*PCHIS*: Private Commercial Health Insurance Scheme.

The objective of the present paper is to depict the role and interests of key stakeholders and how their power to contest proposed reforms has shaped the agenda setting and policy formulation for the proposed NHIS in Uganda. Various terms are used to describe the entities involved, such as actors, players, stakeholders, and interested parties [48]. We use the term ‘stakeholder’ to signify an ‘entity with a declared or conceivable interest or stake in a policy concern’ [49]. This includes individuals, organisations, collectives, and various groups, either formally organised or unorganised. This process of managing stakeholders is a vital factor for achieving health policy change [50,51].

## Methods

The methodology adopted here is a single case study of agenda setting and policy formulation related to the proposed NHIS in Uganda. It involves an analysis of real-life context, the content of proposals and the process, and a retrospective stakeholder analysis using the single instance of NHIS policy development. The choice of research method matched our goal of providing a retrospective analysis of the participants (both individuals and groups) engaged in the NHIS development, examining how action or inaction by the stakeholders influenced this process, and investigating the context in which the process unfolded. Data collection was carried out through literature reviews of published and unpublished documents; these included technical reports, policy briefs, and memos obtained from the MOH plus an analysis of secondary data collected by UNICEF for a survey of NHI in 52 countries [45]. Formal discussions with selected senior health ministry staff and NTF members involved in the design of the scheme were conducted to obtain clarifications, verify events, and obtain background information. Express permission to carry out this research and access all data was granted by the MOH. This study was part of the work program of the Ugandan health sector approved by the government, donors, and all stakeholders as indicated in the second National Health Policy and Health Sector Strategic and Investment plans.

### Stakeholder analysis and design of the NHIS

Although political stability is important [52], the successful design and implementation of health insurance also greatly depends on consensus among the key stakeholders and securing their support for all its elements, including the benefits package, level of contributions, and details of who contributes what proportion of the premiums [53]. The process of stakeholder analysis refers to a methodology for ‘systematically gathering and analysing qualitative information to determine whose interests should be taken into account when developing and/or implementing a policy or program’ [48]. Stakeholder analysis allows policy makers to anticipate the challenges and the relative ease with which the policy can be introduced and understand how to manage the process more effectively. Effective engagement of stakeholders also ensures the sustainability of a reform because it provokes ‘the creation of actors who will defend their new interests in the political process’ [54].

### Stakeholders and their policy positions

The development of the NHIS involved numerous sectors and stakeholders. For example, the MOH identified 55 stakeholders that have in some way shaped the CHI—one of the four component schemes of the NHIS [55]. Our analysis focused on selected stakeholders at the core of the overall NHIS design process (Table 3).

**Table 3:** Partial list of key stakeholders shaping Uganda’s NHIS by group affiliation

**National government actors/ politicians**	**Public sector**	**Private sector**	**Civil society**	**Donors**
i) Parliament (ruling party and opposition)	i) National Social Security Fund (NSSF)	i) Private for profit and non-profit providers	i) NGOs	i) Providing for Health (P4H).
ii) President (executive president)	ii) Insurance Regulatory Authority of Uganda (IRAU)	ii) Private insurance companies	ii) Trade unions and employee groups	ii) Swedish Development Agency
iii) Prime minister	iii) National Planning Authority (NPA)	iii) Private-sector foundation	iii) Health professional associations	iii) UK Department of International Development
iv) Cabinet		iv) Uganda Manufacturers Association		iv) Belgian Technical Cooperation
v) Ministry of Health		v) Community health insurance schemes		
vi) Ministry of Finance, Planning and Economic Development		vi) Federation of Uganda Employers		
vii) Ministry of Public Service				
viii) Ministry of Gender, Labour and Social Development				
ix) Local governments (district and urban authorities)				

We analysed four attributes of NHIS stakeholders: their stated positions; level of influence (power) they held; the level of their interest in reform; and their group or coalition affiliations [48]. Based on these attributes, we categorised stakeholders according to their level of support and degree of political influence. This created a stakeholder matrix or ‘position map’ [51]. The results of this analysis can inform political strategies so as to increase the likelihood of reform being accepted by designing tailored approaches for each stakeholder group. It has been argued that for a health policy to be successful, it needs to achieve ‘synergies between sectors and actors’ [52]. We assessed stakeholder synergies and complex interactions largely through a review of historical events and retrospective contextual research. This partial analysis focused on stakeholders who demonstrated high- or medium-level support on a continuum of political power. Table 4 summarises the dominant positions of the NHIS stakeholders.

**Table 4:** NHIS stakeholders by level of support and influence

	**High support**	**Medium-level support/low opposition**	**High opposition**
**High influence**	i. Parliament	i. Trade unions	i. National Social Security Fund (NSSF)
	ii. Prime minister	ii. Federation of Uganda Employers	
	iii. Cabinet	iii. Uganda Manufacturers Association	
	iv. Ministries (MOH, MOFPED, MOPS, MOGLSD)	iv. Private sector foundation	
	v. Providing for Health (P4H)		
**Medium-level influence**	i. Insurance Regulatory Authority of Uganda	i. Local governments (district and urban authorities)	-
	ii. IRAU		
	iii. National Planning Authority		
**Low influence**	i. Private for-profit providers	i. NGOs & religious medical bureaus	-
	ii. Community Health Insurance Schemes	ii. Donors (bilaterals)	
		iii. Health professional associations	
		iv. Private insurance companies	

Adapted from Roberts et al. 2008 [51].

### Stakeholders offering a high degree of support and having high-level influence

Support of the NHIS by the government was high at both ministerial and cabinet levels. The following provided ongoing support for the NHIS, most notably during the second and third phase of its development: the MOH; Ministry of Finance, Planning and Economic Development; Ministry of Gender, Labour and Social Development; and Ministry of Public Service. Their support was formally expressed through a cabinet minute (‘decree’) in September 2011. Parliament has viewed the NHIS as a means to improve access to quality services and alleviate the suffering of the Ugandan population. A parliamentary sub-committee in April 2012 gave the go-ahead to the NTF to finalise drafting of the NHIS bill for final approval. Two cabinet decrees (2006 and 2011) and the Ruling Party Presidential Manifesto calling for NHI signalled strong support at the cabinet level. The prime minister, who chairs the cabinet, made a public statement, expressing his support for the NHIS. Even members of the opposition party voiced their support [47]. As of late 2011, the various political actors remained united behind the NHIS as an all-party achievement. However, the president has not publicly backed the latest NHIS plan. This may signal the awareness of the executive branch that some stakeholders, such as private-sector insurers, still harbour concerns that the NHIS could negatively affect growth of the private insurance market. P4H worked through the MOH to provide technical support for the NHIS design process as well as implement criteria and strategies. Through P4H, the World Bank participated in the NTF. The bank catalysed stakeholders’ engagement, such as by funding a study tour to three East Asian countries (Thailand, Vietnam, and China) so that cabinet members could examine SHI schemes. The WHO country office also participated in the NTF to advise on design issues and support sensitization of parliament and cabinet members. One critical area of WHO support was for a combined team from the ministries of health and finance to develop robust estimates for NHIS revenue and expenditure using SimIns software [20].

### Stakeholders offering a high degree of support and having medium-level influence

As an agency with vested power to determine priority areas for national development planning, the National Planning Authority (NPA) produced the National Development Plan 2010/11–2014/5, wherein establishing an NHIS was set forth as a core area of health sector reform [29]. As a member of cabinet, the NPA chairperson also provided strong support for the NHIS. The Insurance Regulatory Authority of Uganda (IRAU), which oversees all insurance operations in the country including health insurance, was an important stakeholder in influencing the NHIS design and planned implementation. As a member of the NTF, the IRAU played a key role in drafting the cabinet memo on NHIS principles that was approved in 2011.

### Stakeholders offering a high degree of support and having low-level influence

CHI schemes are managed by private organisations, which have only limited influence on proposed government reform, though their support for NHIS was strong. For them, the NHIS represented an opportunity for growth and expanded enrolment in CHI schemes. The CHI schemes influenced policy through their umbrella network, the Uganda Community-Based Health Financing Association, which lacks formal opportunities for influencing NHIS policy beyond membership of the NTF.

### Stakeholders offering medium-level support and having a high degree of influence

The private sector, including the Uganda Manufacturers Association and Private Sector Foundation, wields substantial influence. This is largely through the Presidential Investment Forum, in which the private sector advises the president on market development, employment, and taxation policy. However, the proposed SHI bill generated strong negative reactions from non-MOH stakeholders, primarily in the private sector, who believed that SHI would displace private health insurance schemes [40]. Employers, specifically manufacturers, were concerned that the obligation to pay a share of the insurance premium for enrolled employees would increase production costs [17]. Formal-sector employees and employers were apprehensive about the capacity of the government to manage the SHI Fund: this was the result of a history of public funds mismanagement [17,31], most notably cases involving the Global Alliance for Vaccines and Immunization and the National Social Security Fund [56,57]. In addition, concerns were raised about a potential worsening of the already poor-quality services because the costs to meet increased demand for services would exceed the additional resources forecasted to be generated by SHI [19].

Because of these concerns from several influential stakeholders, the president halted the process and called for more dialogue between the MOH and the private sector. Key actors in the health sector thought that this was simply a health-sector issue and did not adopt a multi-sectoral approach and engagement among all stakeholders. The MOH and NTF were able to garner some support from the private sector by negotiation. Though some concerns remain in the private sector, for example about NHIS increasing production costs and the risk of business failure, the position of the NHIS is now closer to that of a supporting coalition than it was during the second phase of its development. A second influential group is that of private insurers, who are concerned about losing their market share to the NHIS [58]. A recent study showed that only 36% of surveyed employers would continue subscribing to private health insurance schemes after implementation of the NHIS [31]. Responding to their interests rather than the position they took on NHIS, the MOH has agreed on a strategy of liberalising the health insurance industry. The trade unions have special seats in the national parliament, which allow them to participate in policy debates. Trade unions are concerned about the proposed 4% payroll deduction from workers’ pay [17]. However, discussions are continuing with the unions and the NSSF about whether the 4% can be part of the NSSF contribution made by employees. It should be noted that some large employers would fully cover the 8% contribution because of existing collective bargaining agreements mandating health insurance coverage.

### Stakeholders offering medium-level support and having medium-level influence

Local governments wanted the scheme to be implemented. However, they had strong concerns over the potential burdens that would be imposed in terms of administration as well as the level of their contributions to its financing [59].

### Stakeholders offering medium-level support and having low-level influence

Health professional associations, such as the Uganda Medical Association and the Nurses and Midwives Association, questioned whether the NHIS could have the intended impact of increased access to quality care and improved population welfare [60]. Though the media could potentially play an influential role by raised public awareness of the NHIS, the main media outlets had neither adopted a position about establishing the NHIS nor focused much coverage on the proposed health reform legislation.

### Stakeholders offering a high degree of opposition

During Phase I, the management of the NSSF was also opposed to the establishment of an SHI scheme. That NSSF management felt that it was the best-qualified institution to run an SHI scheme [61], and it viewed the creation of an SHI as threatening its authority and influence.

## Results and discussion

### Health financing

Households contribute over 40% of THE funds, which results in great inequalities in general health and access and use of healthcare services because it disproportionately constrains poorer people from accessing the necessary care. This situation also increases the incidence of catastrophic expenditure, thereby increasing the level of poverty [62]. Current health-financing trends provide the option of a pooled mechanism for health insurance in the case of limited government funding.

### Stakeholder engagement

Roberts et al. proposed that to create alliances for supporting new policies and introducing health reform, it is necessary to develop political strategies to manage the power and positions of stakeholders. Some political strategies seek to change the position of stakeholders: some aim for redistribution of power among the stakeholders; some pursue a change in the number of players or their group affiliations; and some seek to change the perception of the problem or the solution [51]. The private sector was vehemently opposed to the establishment of virtually any NHI arrangement until the minister of state responsible for health insurance addressed them in a collective forum during Phase II. Negotiations were conducted in the presence of a neutral arbitrator from the World Bank. Among the grievances of private-sector stakeholders were their exclusion from NHIS planning consultations, the possibility of increases in the cost of production, and lack of representation at the planning level [17]. The private sector demanded a voice in selecting NTF members and being guaranteed participation in NHIS design and management [58]. In addition, the private sector desired liberalisation of the scheme’s operations. The negotiations led to a more comprehensive NTF that represented all stakeholders, with provisions for liberalising the insurance plan then being adopted in a new NHIS draft bill.

The private-sector representatives attend NTF meetings and carried out sensitisation missions for other stakeholders. Similarly, private commercial insurers were concerned about business being lost to the NHIS, and they maintained a strong involvement in the NHIS design process. The previous management of the NSSF, which was also opposed to the NHIS, was replaced. The new management adopted the view that a comprehensive insurance scheme was desirable, and it demonstrated its commitment by agreeing to sit on the NTF. Civil society groups did not take on a strong position on the NHIS and remained largely passive, possibly through a lack of awareness of the significance and implications of the NHIS for the general public. Furthermore, non-state providers of services were more concerned about ensuring sufficient financing to maintain existing services than examining ways to expand those services. Some concerns, such as those related to the impact on fair access to and quality of healthcare, were shared among stakeholders with different levels of support and influence. NGOs and donors were both concerned whether the NHIS could deliver on its promise of better-quality and more equitable healthcare [60]. The change of position of such stakeholders as the NSSF and private insurers illustrates the key characteristics of stakeholders on how support for a policy can shift over time, and in this case such shifts provided opportunities for developing NHIS policy.

### Additional considerations

The stability of the Ugandan political system, with the president entering his 26th year in office, offers a strong advantage for the NHIS. Recent health reform success cases (e.g., Bangladesh, Kyrgyzstan, and Ethiopia) emphasise the importance of strong bureaucracies and a stable political system for effective implementation of such reform [52]. Conversely, the impetus for the NHIS was strongly associated with democratic reforms in Uganda, in which the NRM won multi-party elections in 2006 and again in 2011.This is similar to the cases of Ghana [2] and Tanzania [4], where health insurance was included in the election manifesto of the ruling party and introduced both to meet the expressed wishes of the electorate and ensure a presidential legacy.

The role of the presidency in Uganda can be compared to that of Kenya during the development of the National Social Health Insurance Fund (NSHIF) law [63,64]. An inter-sectoral taskforce was established to prepare a national strategy and legislation as a first step towards formulating the NSHIF. In 2004, the Kenyan parliament passed the NSHIF bill, but the president did not accede to it, partly because he had not been fully consulted on the bill [65]. This clearly indicates the relevance of stakeholder analysis and engagement at the highest level; Uganda and other countries could learn from this experience.

The process in Uganda can also be contrasted with development of the Ghanaian NHIS. The situation in Ghana posed a different type of challenge, involving stakeholders with dissimilar political agendas and ideological convictions. NHI was pioneered by the then ruling party, the New Patriotic Party (NPP), which used the NHIS as an election campaign plank. Shortly after the elections, the NPP appointed a taskforce to spearhead the design of the NHIS. Although the taskforce prepared a technically sound strategy, it lacked the political support of some of the key stakeholders because it was regarded as an initiative of the former government, whereas the aim of the new government and its key allies had been to put in place a complete overhaul of the public system [2]. This led to significant difficulties and delays in establishing the NHIS policy and its passage through parliament. It also further polarised two sets of stakeholders and created a division that could have been prevented had the task force conducted a stakeholder analysis and used it to respond to the concerns of influential players. The Ghanaian case highlights the potential value of stakeholder analysis in guiding and managing health reform [2]. It further emphasises the need ‘to promote better dissemination, understanding and use of analytical frameworks on the political economy of reform in developing countries’ [66]. The experience in Ghana, as in Uganda, showed that high-level political commitment, popularity of the proposed reform, and sound technical analysis may be insufficient to avoid implementation difficulties. The political party and the then president’s support was clear in Ghana, though this has yet to be seen in Uganda. In Ghana, the then ruling party and president also wanted to show the electorate that their party had fulfilled an election pledge before the next elections. Greater attention and focus needs to be placed on recognising, analysing, and managing the political interests of key stakeholders during the policy process.

As the Ugandan health sector makes continued efforts in designing the scheme, the current government still faces the policy challenge of increasing health expenditure as a proportion of public financing from the current 7% to meet the Abuja target of 15%. Indeed, only five countries—Botswana, Rwanda, Zambia, Madagascar, and Togo—have attained that target [67]. Establishing the NHIS would require greater public outlays to cover the projected reduction in OOP payments.

We recommend further research to elucidate additional details on the related technical, substantive, and operational factors towards clarifying the major reasons for preventing an NHI scheme from being established in Uganda—as compared with the East African countries that have advanced further in this area. Such studies could provide greater information on the enabling and constraining factors, such as the scope of services included in insurance benefit packages, the level of private contributions, and the level of coverage provided to various sub-populations, particularly the poor, informal-sector workers, and other groups not covered by private insurance plans.

## Conclusions

Uganda provides an interesting case study for exploring how planning for NHI has evolved since 1995 and moved to the current phase of near-implementation. The design of the NHIS was characterised by small-scale, gradual changes and adjustments during all three phases of development. Despite political will in government, resistance by various stakeholders played an important role in constraining the pace of the development process as well as in shaping the design of the proposed NHIS.

The study points out how a policy-making process characterised by negotiation, bargaining, and adjustment of policy actions responded to stakeholders’ aspirations and positions. Among other ways, the stakeholders influenced the policy makers in changing the design and name of the proposed health-financing reform from SHI to NHI. Stakeholders were also influential in changing the design from a single to multiple schemes and in the immediate incorporation of the informal sector in the early design and operation of the scheme.

In line with its neighbours, Uganda set out to establish an NHIS to provide for more equitable access to services as well as for a more durably financed health sector. The initial proposal received a low level of support, mainly because the plan was developed without taking into consideration key stakeholders and thus failed to meet their concerns. The preparation and planning for NHI in Uganda was overly concerned with policy content, and very little thought was given to stakeholder engagement. Even the study tours to three East Asian countries failed to identify the general process by which policy reform was negotiated, stakeholders analysed, and political institutions engaged and managed. The initial phase had a limited knowledge base and minimal available research to provide guidance and practical examples for the political economy in establishing NHI. Lacking a good evidence base, the sponsors of the NHIS relied largely on technical solutions and past experience with other health reforms. The case of the NHIS in Uganda thus offers several valuable lessons:

•It is vital to undertake a comprehensive stakeholder analysis as part of any substantive health-sector reform to identify, address, and overcome concerns before they harden into inflexible opposition. An intensive, detailed stakeholder analysis during the design process could pinpoint rising issues or threats, minimise obstacles to passage, build coalitions, and channel information and resources to promote and sustain reform implementation [49,51].

•The positions of stakeholders can be influenced to shape the direction in which they develop over time. Thus, policy makers and implementers need to consider periodic re-categorisation of stakeholders to capture emerging positions and shifting power dynamics as well as sustain their continued engagement. If stakeholder analysis is not re-evaluated at regular intervals, it can slow down or halt the policy design and development process—even when there is strong support from senior executive and legislative representatives.

•The private sector represents an important stakeholder in health-financing reforms, and its role needs to be carefully considered. Comprehensive feasibility and actuarial studies of the health insurance scheme need to be complemented by broader political economy analyses and social impact assessments that specifically examine the potential effects of SHI on employment and investment in the private sector.

•A situational analysis of health-sector policies and recent health reforms may need to be conducted to identify potential conflicts with the proposed NHI plan. Launched after major political change and against the backdrop of abolition of user fees, the NHIS faces additional challenges that overlap with technical issues, stakeholder management, and the common apprehension that often surrounds major health reforms. It is necessary to address these challenges. This will entail reconciling the views of two different sets of stakeholders on how to overcome the financial barriers to access: those advocating user fee abolition to achieve universal healthcare; and those advocating the imposition of some level of contribution for insurance coverage as the vehicle for universal healthcare. For example, Kenya and Tanzania had to reintroduce user fees before implementing their NHI schemes [4,64,68]. The government of Uganda may need to re-evaluate the impact on changes in the level of community financing of services in the current environment of abolishing user fees before pressing on with stakeholder discussions and NHIS implementation [6,64].

A growing body of literature supports the argument that an analysis of stakeholders and the policy arena is highly relevant to health-policy reform and represents an important first step in developing plans for NHI. This paper points out how policy making is a complex process with an unstable and rapidly changing context. The use of stakeholder analysis in predicting and managing the future is time limited, and it is desirable that it be supplemented by other policy analysis approaches, such as the Delphi method [69]. Our results from this Uganda case study add to the body of evidence and offer useful information to policy makers and those tasked with designing and implementing SHI in low- and middle-income countries or similar settings.

## Abbreviations

CHIS: Community health insurance scheme; HSSIP: Health sector strategic and investment plan; IRAU: Insurance regulatory authority of Uganda; MOFPED: Ministry of finance, planning and economic development; MOGLSD: Ministry of gender, labour and social development; MOH: Ministry of health; MOPS: Ministry of public service; NDP: National development plan; NGO: Non government organisation; NHIS: National health insurance scheme; NPA: National planning authority; NSSF: National social security fund; NTF: National task force; OOP: Out-of-pocket expenditure; PCHIS: Private commercial health insurance scheme; P4H: Providing for health; NRM: National resistance movement; SHI: Social health insurance.

## Competing interests

The authors declare that they have no competing interests.

## Pre-publication history

The pre-publication history for this paper can be accessed here:

http://www.biomedcentral.com/1472-6963/13/357/prepub
